# Bridging speech and sight: white matter anatomy in ticker-tape synaesthesia

**DOI:** 10.1093/braincomms/fcaf316

**Published:** 2025-08-29

**Authors:** Romain Delsanti, Fabien Hauw, Romain Lahbari, Florence Bouhali, Laurent Cohen

**Affiliations:** Inserm U 1127, CNRS UMR 7225, Sorbonne Universités, Institut du Cerveau, ICM, Paris 75013, France; Inserm U 1127, CNRS UMR 7225, Sorbonne Universités, Institut du Cerveau, ICM, Paris 75013, France; AP-HP, Hôpital de La Pitié Salpêtrière, Fédération de Neurologie, Paris 75013, France; Inserm U 1127, CNRS UMR 7225, Sorbonne Universités, Institut du Cerveau, ICM, Paris 75013, France; Aix Marseille University, CNRS, CRPN, Marseille 13003, France; Inserm U 1127, CNRS UMR 7225, Sorbonne Universités, Institut du Cerveau, ICM, Paris 75013, France; AP-HP, Hôpital de La Pitié Salpêtrière, Fédération de Neurologie, Paris 75013, France

**Keywords:** synaesthesia, reading, structural connectivity

## Abstract

Ticker-tape synaesthesia is an informative yet little studied developmental condition in which persons see automatically and vividly in their mind’s eye the written form of the spoken words they are hearing. Ticker-tapers show an over-activation and a functional over-connectivity of core regions of the left-hemispheric reading system: the posterior superior temporal and supramarginal gyri (pSTG/SMG), where speech is processed and interfaced with vision, and the Visual Word Form Area (VWFA), which supports orthographic representations in the occipitotemporal cortex. We predicted that synesthetes should show increased anatomical connectivity between these regions. We scanned 17 synesthetes and 17 matched controls with diffusion-weighted MRI, and used probabilistic tractography to compare the density of streamlines between groups. We found that ticker-tapers had a higher streamline density in the white matter underlying the SMG, and connecting the SMG and the mid and posterior STG. We propose that those increased white matter connections at the temporoparietal junction and towards the VWFA boost the top-down influence of phonology on orthography, giving rise to the ticker-tape phenomenology. More generally, while atypical anatomical connectivity may be detrimental to the acquisition of culture-dependent abilities, as in dyslexia, it may also underlie a gain of function, as illustrated by ticker-tape synaesthesia.

## Teaser

Delsanti *et al*. reported that ticker-tape synaesthesia, a developmental condition in which people see automatically the written form of the words which they are hearing, is associated with increased anatomical connectivity within the brain reading network. These results offer new insights into the neural bases of language development and synaesthesia.

## Graphical Abstract

**Graphical Abstract:**
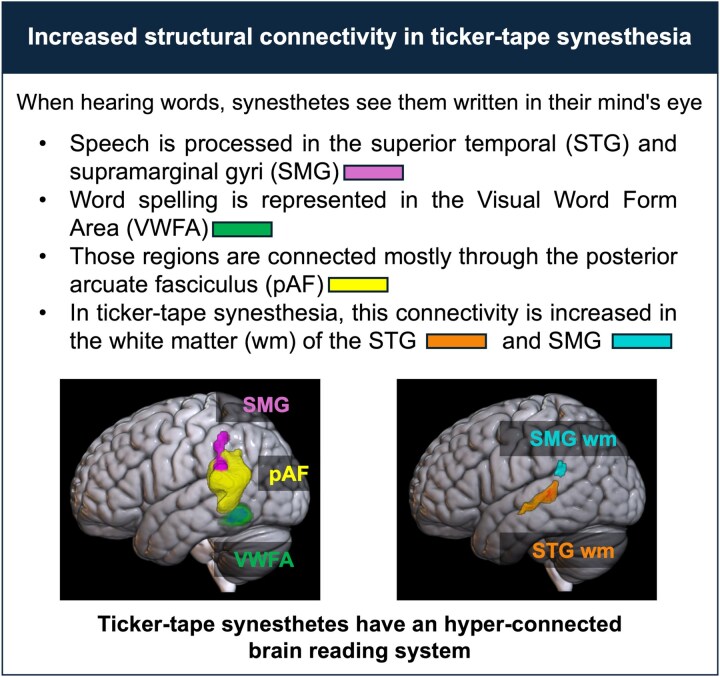

## Introduction

The defining feature of literacy is the ability to translate rapidly and accurately between sounds and strings of letters. This ability is supported by changes that occur during reading acquisition in the auditory cortex, where speech sounds are processed, in the visual cortex, where letters and letter combinations are recognized, and in white matter pathways connecting them.^1^ The brain substrate on which such changes operate shows individual variability, partly explaining the variability of the eventual reading phenotype, which covers the range of typical reading,^2^ but also developmental dyslexias^3^ and reading-related synesthesias such as grapheme-colour or ticker-tape synesthesias.^4^

Schematically, the core regions supporting the cross-modal integration of sounds and letters in the left hemisphere include the left temporoparietal junction for sound processing, the ventral occipitotemporal cortex for letters and graphemes recognition, and connections between them through the arcuate fasciculus. More specifically, speech sounds are processed in the posterior superior temporal cortex (pSTG) and the adjacent supramarginal gyrus (SMG). While other aspects of phonology are processed in more anterior temporal regions, the pSTG/SMG acts as an interface between speech sounds and other modalities, for instance during lip-reading or speech repetition.^5^ During reading and spelling, it is particularly involved in phonology-based translation between sounds and letters.^6^ As for the visual recognition of ordered letters, it is achieved in the Visual Word Form Area (VWFA), a reproducible sector of the left ventral occipitotemporal cortex.^7,8^ The emergence of the VWFA is tightly correlated with the mastery of reading,^9^ lesions result in selective reading impairments,^10^ and its functional properties are finely tuned to the specifics of the familiar scripts.^11^

The links between the pSTG/SMG and the VWFA predate reading acquisition. Thus in Feng *et al*.^12^ children were presented with short blocks of written words and other types of pictures, while detecting an odd-ball target. Before reading acquisition, the VWFA did not show word-specific activation, but was already functionally connected to left-hemispheric spoken language areas including the pSTG/SMG. Those links were further strengthened with the improvement of reading ability.

This functional connection is particularly engaged during phonology-based reading.^8^ Anatomically, the exchange of information between the VWFA and the pSTG/SMG is supported by the posterior branch of the arcuate fasciculus (pAF), whose fractional anisotropy is positively correlated with literacy.^13^ Conversely, anatomical features of the left arcuate fasciculus are reproducibly abnormal in dyslexia.^14^

We recently showed that the pSTG/SMG and the VWFA are key players in ticker-tape synaesthesia (TTS), a developmental condition affecting the reading system.^15,16^ Persons with TTS perceive automatically and vividly in their mind’s eye the written form of any speech which they are hearing^17^ and they perform better than typical individuals on tasks involving orthographic mental imagery.^18^ In ticker-tape synesthetes, during speech perception, the pSTG/SMG and the VWFA are both over-activated and functionally over-connected,^19^ supporting the hypothesis that TTS results from an increased top-down phonological influence on orthographic representation.^20^

We report the first study of the connectional anatomy in TTS. Based on the above considerations, we predicted that synesthetes should show increased anatomical connectivity between the left pSTG/SMG and the VWFA, through the pAF.

## Materials and methods

### Participants

We recruited participants by broadcasting a short description of TTS through email lists and social networks, targeting particularly groups of students and university members, and groups devoted to synaesthesia, psychology and neuroscience. We thus recruited 17 ticker-tape synesthetes, plus 17 matched controls. All participants were native French speakers, right-handed according to the Edinburgh Inventory and had no history of neurological or psychiatric disorders. Participants were matched one-to-one between groups in gender (14 females/3 males), in age (synesthetes: mean 39.7 years ± 13.9, standard deviation; controls: mean 38.6 ± 14.4 years; Student’s *t*-test: *P* = 0.83) and in education level. The research was approved by the institutional review board of the INSERM (protocol C13-41), and all participants provided informed written consent in accordance with the Declaration of Helsinki.

### Images acquisition and processing

#### Acquisition

Diffusion-weighted imaging (DWI) data were acquired on a Siemens 3T MAGNETOM Prisma scanner, with a 64-channel head coil (TR/TE/flip angle = 320 ms/89 ms/78°, voxel size = 1.5 × 1.5 × 1.5 mm^3^). A total of four sequences (398 volumes) were acquired for each participant, two in both phase encoding directions (anterior-posterior AP and posterior-anterior PA) with 98 and 99 uniformly distributed directions including multiple shells (b values = 1500 s/mm^2^ and 3000 s/mm^2^). Twenty-eight volumes with no diffusion gradient (b = 0 s/mm^2^) were acquired along the session. Acquisition time was 20 min. We acquired 92 contiguous axial slices with a whole-head coverage (in-plane matrix 140 × 140; field-of-view 210 × 210 mm; slice thickness 1.5 mm).

#### Processing

The DWI images were processed using the MRtrix software^21^ to denoise and remove Gibbs’ artefacts. We applied top-up and eddy current correction^22^ as implemented in the FMRIB Software Library (FSL).^23^ The susceptibility-induced off-resonance field was estimated from AP-PA image pairs using a method similar to that described in Andersson *et al*.,^24^ in order to correct distortions. Local diffusion parameters for the tractography were estimated using a Markov Chain Monte Carlo sampling with FSL’s BedpostX. We ran BedpostX using the default settings (three fibres modelled per voxel; weight = 1).

Probabilistic tractography was run in FSL using the probtrackx2 command^25^ and the XTRACT pipeline,^26^ to automatically extract all the white matter tracts potentially involved in literacy, plus their right-hemispheric counterpart: the long segment of the arcuate fasciculus (AF), the posterior segment of the arcuate fasciculus (pAF), the inferior longitudinal fasciculus, the uncinate fasciculus and the inferior occipitofrontal fasciculus. This resulted in one map for each tract for each participant, representing the density of streamlines in each voxel divided by the total number of valid streamlines. A group mask was created for each tract, by normalizing individual density maps to the MNI space, averaging them, thresholding the average density map (density > 5.10^−4^), then smoothing (6 mm FWHM) this thresholded map, and binarizing it. The same method (with density > 10^−2^) was used to create a mask of the connections of the SMG region which we identified in the first analyses.

### Statistical analysis

Within each mask, we compared the voxelwise density of streamlines between synesthetes and controls with age and gender as covariates. We used the FSL ‘randomise’ command, a tool for nonparametric permutation inference, performing 5000 permutations to estimate the null distribution of differences between groups. We then applied a statistical threshold of voxelwise *P* <0.001. Finally, in order to correct for multiple comparisons, we applied Threshold-free Cluster Enhancement (TFCE) which in essence finds significant differences by combining the strength and the spatial extent of signals—without needing to set an arbitrary threshold—and gives corrected significance values for every voxel (*P* < 0.05 corrected within the considered tract). Voxelwise effect size for group comparisons was estimated using Cohen’s d index.

### Functional regions of interest

**Figure 1:**
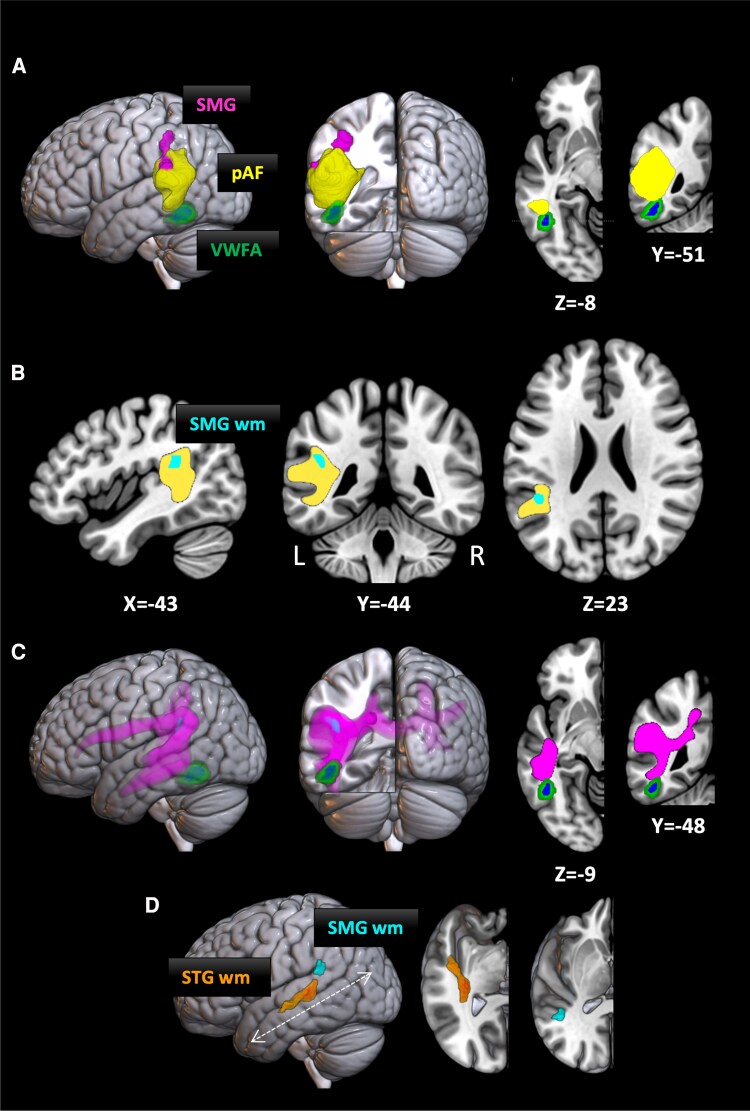
**Regions of increased anatomical connectivity in TTS compared to controls.** (**A**) Lateral and posterior views of the left-hemispheric supramarginal gyrus (SMG, purple), the Visual Word Form Area (VWFA) functionally defined by its selectivity for written words (green) and by its increased activation in synesthetes during speech perception (blue) and the posterior segment of the arcuate fasciculus (pAF, yellow). Axial and coronal slices illustrate the direct contact between the pAF and the VWFA. (**B**) Slices showing a white matter region underlying the SMG, in which the density of streamlines was higher in synesthetes than in controls (cyan; TFCE corrected *P* < 0.05, mean Cohen’s d = 1.18), superimposed on the pAF (yellow). (**C**) Lateral and posterior views showing the SMG region of increased streamline density (SMG wm, cyan) and its white matter connections (purple). Axial and coronal slices show the contact between those connections and the VWFA. (**D**) Lateral view of the regions of increased streamline density in synesthetes, underlying the SMG (SMG wm, cyan) and the superior temporal gyrus (STG wm, orange; TFCE corrected *P* < 0.05, mean Cohen’s d = 1.26). Right: Superior views of oblique cuts parallel to the axis of the temporal lobe (dashed arrow).

The VWFA and the SMG are represented in Fig. 1 on the basis of functional MRI activation from Hauw *et al*.^19^ The VWFA is represented by two precisely overlapping clusters. The green cluster corresponded to stronger activation by written words than by other types of images (faces, houses, tools), the standard defining feature of the VWFA. Activations by this contrast did not differ between synesthetes and controls, and the cluster shown in Fig. 1 results from pooling both groups (voxelwise *P* < 10^−5^ and clusterwise *P* < 0.05 family-wise error corrected). The blue VWFA cluster and the purple SMG cluster were activated in synesthetes more than in controls during speech perception (voxelwise *P* < 0.001 and clusterwise *P* < 0.05 family-wise error corrected, masking by activation in synesthetes relative to baseline at voxelwise *P* < 0.001).

## Results

We acquired DWI data in 17 adult ticker-tape synesthetes, plus 17 matched controls. Our primary prediction was that the left pAF, which links the pSTG/SMG to the VWFA, would show increased connectivity in synesthetes. To assess this prediction, we defined a common pAF template based on the data from both groups. We verified that the pAF reached up to the SMG dorsally, and that ventrally it contacted the VWFA, which was functionally defined as described in the Methods (Fig. 1A). We then compared the density of streamlines in the pAF between groups, and found a higher density in synesthetes than in controls in the white matter underlying the SMG (Fig. 1B; MNI coordinates of the centre of mass: *X* = −46, *Y* = −42, *Z* = 23; effect size: mean Cohen’s d = 1.18).

To assess the specificity of this finding, we applied the exact same method to the other white matter fascicles potentially involved in reading (see ‘Materials and methods’), and to their right-hemispheric counterparts. We found no difference between groups in streamline density in any of those fascicles.

To better understand the situation of this SMG region of increased connectivity in the letter-sound integration network, we used it as a seed for identifying its full tractogram (Fig. 1C). It was connected to the ventral occipitotemporal cortex, including the VWFA, to the superior and middle temporal gyri, and to frontal regions. It was also connected to homologue right-hemispheric areas through the corpus callosum. Within this unbiased tractogram, we again compared the density of streamlines across groups. Synesthetes showed increased density in the white matter of the mid and posterior left STG (Fig. 1D; MNI coordinates of the centre of mass: *X* = −40, *Y* = −27, *Z* = 0; effect size: mean Cohen’s d = 1.26). None of the analyses showed stronger connections in controls than in synesthetes.

## Discussion

Synesthetes showed a selective increase in fibre density in the white matter lying beneath the left SMG and STG. This may constitute the anatomical substrate of the increased functional connectivity that was observed between the left pSTG/SMG and the VWFA in TTS.^19^ As predicted, the first difference between groups was found in the left pAF, a fascicle whose anatomical structure correlates with reading proficiency and with reading-related activation in both the pSTG and the VWFA, across individuals of varying literacy.^13^ No difference was found between the groups in the other white matter tracts tested. We found a second locus of increased fibre density in the white matter linking the pSTG cortex and the SMG. While the respective roles of the pSTG and the SMG in phonological processes are difficult to distinguish,^27^ one may speculate that their connections convey phonological information extracted in the STG cortex to the SMG, where it is interfaced with orthography and influences the VWFA through the pAF.

The directionality of the influence between the pSTG/SMG and the VWFA remains hypothetical, as diffusion imaging is uninformative on this issue. At present, there is no evidence that synesthetes would show a phenomenon reciprocal to TTS (e.g. increased inner speech during reading), suggesting that the increased anatomical connectivity may underlie a unidirectional increase of the influence of phonology on orthographic representations. In a single-case study of TTS, using dynamic causal modelling (DCM), we found indications that speech was driving from top-down the regions that support TTS, while temporally reversed speech, which does not trigger synaesthesia, had an inhibitory effect.^20^ Further studies employing functional time-resolved imaging such as MEG are necessary to assess this hypothesis.

To conclude, any inference from anatomical differences to causal implication should be cautious, as differences in practice and expertise may cause changes to anatomical connections, for instance for motor skills.^28^ In the case of reading, there is actually evidence of a reciprocal causality: the VWFA shows a specific pattern of anatomical connectivity already before reading acquisition,^29^ and reciprocally, reading improves its anatomical connectivity through the arcuate fasciculus.^13^ Evidence from previous work suggests that ticker-tape synesthetes perform better than controls in some tasks requiring orthographic working memory, but do not differ in their mastery or practice of spoken and written language.^18^ One may therefore propose that the increased connectivity which we observed figured among the causes of TTS development. Atypical anatomical connectivity may thus be detrimental to the acquisition of culture-dependent abilities, such as in dyslexia, but may also cause a potential gain of function, as illustrated by ticker-tape synaesthesia.

## Data Availability

Anonymized data and codes are available at https://doi.org/10.5281/zenodo.15664146.
